# Nutritional Risk, Health Outcomes, and Hospital Costs Among Chinese Immobile Older Inpatients: A National Study

**DOI:** 10.3389/fnut.2021.758657

**Published:** 2021-12-10

**Authors:** Hongpeng Liu, Baoyun Song, Jingfen Jin, Yilan Liu, Xianxiu Wen, Shouzhen Cheng, Stephen Nicholas, Elizabeth Maitland, Xinjuan Wu, Dawei Zhu, Wei Chen

**Affiliations:** ^1^Department of Nursing, Chinese Academy of Medical Sciences-Peking Union Medical College, Peking Union Medical College Hospital, Beijing, China; ^2^Department of Nursing, Henan Provincial People's Hospital, Zhengzhou, China; ^3^Department of Nursing, The Second Affiliated Hospital Zhejiang University School of Medicine, Hangzhou, China; ^4^Department of Nursing, Wuhan Union Hospital, Wuhan, China; ^5^Department of Nursing, Sichuan Provincial People's Hospital, Chengdu, China; ^6^Department of Nursing, The First Affiliated Hospital, Sun Yat-sen University, Guangzhou, China; ^7^Australian National Institute of Management and Commerce, Eveleigh, NSW, Australia; ^8^School of Economics and School of Management, Tianjin Normal University, Tianjin, China; ^9^Guangdong Institute for International Strategies, Guangdong University of Foreign Studies, Guangzhou, China; ^10^Newcastle Business School, University of Newcastle, Newcastle, NSW, Australia; ^11^School of Management, University of Liverpool, Liverpool, United Kingdom; ^12^China Center for Health Development Studies, Peking University, Beijing, China; ^13^Department of Clinical Nutrition, Chinese Academy of Medical Sciences-Peking Union Medical College, Peking Union Medical College Hospital Beijing, China

**Keywords:** nutrition risk, mortality, costs, length of stay, immobility, older inpatients

## Abstract

**Purpose:** Evidence of the impact of nutritional risk on health outcomes and hospital costs among Chinese older inpatients is limited. Relatively few studies have investigated the association between clinical and cost outcomes and nutritional risk in immobile older inpatients, particularly those with neoplasms, injury, digestive, cardiac, and respiratory conditions.

**Methods:** This China-wide prospective observational cohort study comprised 5,386 immobile older inpatients hospitalized at 25 hospitals. All patients were screened for nutritional risk using the Nutrition Risk Screening (NRS 2002). A descriptive analysis of baseline variables was followed by multivariate analysis (Cox proportional hazards models and generalized linear model) to compare the health and economic outcomes, namely, mortality, length of hospital stay (LoS), and hospital costs associated with a positive NRS 2002 result.

**Results:** The prevalence of a positive NRS 2002 result was 65.3% (*n* = 3,517). The prevalence of “at-risk” patients (NRS 2002 scores of 3+) was highest in patients with cardiac conditions (31.5%) and lowest in patients with diseases of the respiratory system (6.9%). Controlling for sex, age, education, type of insurance, smoking status, the main diagnosed disease, and Charlson comorbidity index (CCI), the multivariate analysis showed that the NRS 2002 score = 3 [hazard ratio (HR): 1.376, 95% CI: 1.031–1.836] were associated with approximately a 1.5-fold higher likelihood of death. NRS 2002 scores = 4 (HR: 1.982, 95% CI: 1.491–2.633) and NRS scores ≥ 5 (HR: 1.982, 95% CI: 1.498–2.622) were associated with a 2-fold higher likelihood of death, compared with NRS 2002 scores <3. An NRS 2002 score of 3 (percentage change: 16.4, 95% CI: 9.6–23.6), score of 4 (32.4, 95% CI: 24–41.4), and scores of ≥ 5 (36.8, 95% CI 28.3–45.8) were associated with a significantly (16.4, 32.4, and 36.8%, respectively) higher likelihood of increased LoS compared with an NRS 2002 scores <3. The NRS 2002 score = 3 group (17.8, 95% CI: 8.6–27.7) was associated with a 17.8%, the NRS 2002 score = 4 group (31.1, 95% CI: 19.8–43.5) a 31.1%, and the NRS 2002 score ≥ 5 group (44.3, 95% CI: 32.3–57.4) a 44.3%, higher likelihood of increased hospital costs compared with a NRS 2002 scores <3 group. Specifically, the most notable mortality-specific comorbidity and LoS-specific comorbidity was injury, while the most notable cost-specific comorbidity was diseases of the digestive system.

**Conclusions:** This study demonstrated the high burden of undernutrition at the time of hospital admission on the health and hospital cost outcomes for older immobile inpatients. These findings underscore the need for nutritional risk screening in all Chinese hospitalized patients, and improved diagnosis, treatment, and nutritional support to improve immobile patient outcomes and to reduce healthcare costs.

## Introduction

Many older adults suffer from undernutrition that signals a generally poor nutritional status (1–6). The negative health impact of undernutrition is consistent across all age groups, and undernutrition tends to deteriorate during hospitalization, which worsens patient health outcomes, namely, increased frailty, institutionalization, heightened comorbidity, loss of independence, reduced quality of life, higher mortality, and increased hospital costs (4–9). Undernutrition in older adults owing to the lack of intake or uptake of nutrition leads to altered body cell mass and body composition, diminished physical and mental function, and impaired clinical outcomes from the disease (10, 11). We also know that age-related pathophysiological, psychosocial, and pharmacological factors determine changes in dietary habits, and the intake and use of nutrients, leading to specific nutritional deficits (12).

The worldwide prevalence of undernutrition among older hospital patients ranges from 30 to 50% of all admissions (10, 13–16), mainly due to deficiencies in the early assessment, identification, and adequate management of “at-risk” undernutrition patients. The need for comprehensive nutritional screening programs has been widely acknowledged (15), with the European Society for Parenteral and Enteral Nutrition (ESPEN) (17) recommending the Nutrition Risk Screening (NRS 2002) should be used to screen for undernutrition for all hospitalized patients. Several nutritional assessment tools, namely, the NRS 2002 and Short-Form Mini Nutritional Assessment, have been proposed as instruments to identify nutritional risk among hospitalized patients in China (14, 18). Unfortunately, nutritional risk screening is not performed in many Chinese hospitals, with mandatory nutritional risk screening only conducted in some, but not all, large-scale national, provincial, and municipal 500+ bed tertiary hospitals (19).

With the largest population in the world, China has a numerically large number of adults aged 65 years and older, with the ratio of non-working old age to working-age adults growing (20). The undernutrition risk, or high prevalence of undernutrition, in the growing older age population, points to a formidable healthcare burden (20, 21). Immobility is the main cause of deficient nutrient consumption among the elderly, where immobility decreases the ability of myofibrillar proteins to respond to amino acids, so-called anabolic resistance, which contributes to the decline in skeletal muscle mass (22). Older patients may also develop sarcopenia, where the loss of skeletal muscle mass and function is accelerated by immobilization, which frequently is manifested in the form of poor nutritional status (23, 24).

In China, there is a lack of studies addressing the impact of nutritional risk on health outcomes and health costs, especially in older inpatients with immobility. Our national prospective observational cohort study assesses the association between nutritional risk and clinical outcomes and hospital costs among hospitalized immobile older inpatients, and whether the associations differed by sex, age group (60–69, 70+), disease diagnosis, and Charlson comorbidity index (CCI) score.

## Methods

### Study Design and Sample

Supported by the agenda of the National Health and Family Planning Commission to improve the outcomes among older inpatients, the target population is all older adults hospitalized in 25 general hospitals in China. To ensure the representativeness of the study sample, between November 2015 and July 2017, we used a two-stage stratified random sampling design to create a nationally representative sample of patients in China. In the first stage, a simple random sampling procedure was used to select five provinces and Beijing municipality in eastern China (Guangdong province, Zhejiang province, and Beijing municipal city), western China (Sichuan province), and central China (Henan province and Hubei province), a total of six tertiary hospitals enrolled in this stage. In the second stage, 11 secondary hospitals and eight community hospitals were randomly selected from the hospitals attached to these tertiary hospitals.

We collected data on immobile inpatients aged ≥ 65 years old; with basic physiological needs carried out in bed, except for active or passive bedside sitting/standing/wheelchair use for examination; and willingness to provide informed consent. A total of 5,386 participants were enrolled in the study, with follow-ups continuing 90 days after enrolment unless they died in the hospital or relinquished medical treatment.

### Bioethics

The study was approved by the Ethics Committee of Peking Union Medical College Hospital (S-700), and all participants, or their proxies, provided written informed consent before enrolment in the study. Records and information of patients were anonymized and de-identified before the analysis.

### Data Collection

The data were collected by trained and certified registered nurses. To ensure data quality, the research group developed the project survey manual and operation manual. To ensure accurate data collection, all the nurses received systematic training and testing before they recorded information of patients and applied the NRS 2002. They are all proficient in the process of investigation. All questionnaire results were reviewed by the attentive head nurse in each ward to ensure the completeness and correctness of the raw data. Also, the research group established a quality control team, a communication platform based on the WeChat App to guarantee timely feedback. Proxy respondents, usually a spouse or other legal guardian, were interviewed when the patients were incapable of responding to the questions themselves.

### Measurement of Nutritional Risk

According to the European Society for Parenteral and Enteral Nutrition (ESPEN) recommendations (17), Nutrition Risk Screening (NRS 2002) should be used to screen undernutrition in all hospitalized patients. Previous studies also indicated that the NRS 2002 has a high sensitivity (62%) and specificity (93%) and that the NRS score predicts clinical outcomes (18). Even when alternative measures, such as the Short-Form Mini Nutritional Assessment may be more suit for the assessment of the older adult (25, 26), large-scale national, and provincial tertiary hospitals in particular (19) were required to use the NRS 2002 for nutritional risk screening (18). Therefore, this study applied the NRS 2002 among the study participants.

Using NRS 2002, nutritional risk status and disease severity of patients were collected by nursing staff on admission (17). The “nutritional score” was defined by the adequacy of dietary intake due to three different parameters: (i) quartile decrease of estimated oral food intake requirements; (ii) presence of at least 5% weight loss within the previous 1–3 months; (iii) low body mass index (<18.5 kg/m^2^). The NRS 2002 score was calculated by adding the “nutritional score” of 0–3 to the “disease severity score” of 0–3, plus one extra point for “older” patients, who were aged 70 years and older as a subset of all over 65-year-old participants. A total NRS 2002 score ≥ 3 was considered as nutritionally “at-risk,” and the “disease severity score” was categorized as moderate = 3, high = 4, and very high = 5+ (8). NRS 2002 has a good prognostic value for a range of health outcomes, including mortality, with excellent test characteristics (8, 15), and has been validated for the Chinese population (18, 27).

### Outcome Measures

The following outcomes were measured: death (all-cause mortality was recorded at 90 days, including in-hospital deaths, which were verified from death certificates), duration of hospitalization measured by the length of hospital stay (LoS), and hospital treatment costs. Treatment costs were derived from the Hospital Information System (HIS) in each hospital after the enrolled patients died or were discharged from the hospital. The HIS belongs to the financial system of the hospital, which records all the expenses incurred by the patient during their hospital stay.

### Covariates

We collected sociodemographic variables and health-related variables, with the covariates selected based on previous research (8, 14–16, 28). The demographic characteristics included sex, age, education (illiteracy, primary school, junior high school, and high school and above), type of insurance [no insurance; New Cooperative Medical System (29); Urban Resident Basic Medical Insurance (30); and Urban Employee Basic Medical Insurance (31)], smoking status (never, current, and past smokers, which refers to at least 6 months without smoking), and disease diagnosis according to the International Classification of Diseases (ICD)-10 codes (circulatory system, neoplasms, injury, digestive system, respiratory system, and “other”). The CCI provides a reproducible tool to identify patients with multiple chronic diseases in a universally applicable, transparent, and auditable method. CCI measures multiple comorbidities by creating a sum score, weighted according to the presence of 19 comorbid conditions (32, 33). The CCI score was derived from the discharge ICD-10 codes and patient histories obtained from the HIS standardized case report forms. The total CCI score for each patient was categorized into four levels of comorbidity, 0 (none), 1 (moderate), 2 (severe), and 3+ (very severe) (33, 34).

### Statistical Analyses

Statistical analysis was conducted using Stata version 14 for Windows (Stata Corp, College Station, TX, USA). Descriptive results are expressed as mean and SD or as number and percentage. Bivariate analyses were performed using the χ^2^ test or Fisher's exact test for qualitative variables and Kruskal–Wallis test for quantitative variables. Cox proportional hazards models were constructed to determine the association of nutritional risk with mortality and a generalized linear model with a gamma distribution and a log link was used to assess the association of LoS and hospital costs with the NRS 2002 score. The NRS 2002 score was modeled as both a continuous variable and a categorical variable (NRS 2002 <3, 3, 4, 5+). The results were reported as hazard ratios (HRs) in mortality and reported as percentage changes (=exp∧coefficient-1) and 95% CIs in LoS and hospital costs. We adjusted for covariate factors in three stages: (1) we adjusted for age and sex; (2) we added education, insurance, and smoking status; and (3) we additionally adjusted for disease diagnosis and CCI score (the fully adjusted model). To examine the shape of the association between NRS 2002 scores and mortality, LoS, and hospital costs, we conducted a restricted cubic spline analysis. We analyzed whether the association of mortality, LoS, and hospital costs with the NRS 2002 score differed by sex, age group (60–69, 70+), disease diagnosis, and CCI score by separately adding an interaction term to the fully adjusted model. A *P* value of < 0.05 was considered statistically significant.

## Results

### Participant Characteristics

As shown in the baseline sample characteristics in Table 1, 57.5% of patients (3,096/5,386) were men; half (49.9%) were aged 70 years and older; only 18.9% of patients were illiterate; most (80.5%) had insurance; and 70.5% were non-smokers (70.5%). The most frequent diseases were circulatory system diseases (31.5%), others (21.9%), and neoplasms (21.0%), with the proportion of patients with no comorbidities was 27.1%; one comorbidity 26.9%; two comorbidities 23.2%; and three or more comorbidities 22.8%.

**Table 1:** Characteristics of 5,386 immobile Chinese older inpatients concerning NRS 2002 score on admission.

	**Overall**	**NRS <3**	**NRS = 3**	**NRS = 4**	**NRS ≥ 5**	***P*-value**
	**(*n* = 5,386)**	**(*n* = 1,869)**	**(*n* = 1,320)**	**(*n* = 1,005)**	**(*n* = 1,192)**	
Vital status						<0.001
Survived	4,933 (91.6)	1,780 (95.2)	1,215 (92.0)	889 (88.5)	1,049 (88.0)	
Deceased	453 (8.4)	89 (4.8)	105 (8.0)	116 (11.5)	143 (12.0)	
Average length of stay, mean (SD)	17.8 (16.0)	15.0 (10.7)	17.4 (15.2)	20.2 (21.6)	20.8 (17.5)	<0.001
Average hospital cost (Thousands), mean (SD)	52.8 (63.1)	44.8 (45.0)	53.5 (66.1)	56.2 (70.2)	61.9 (75.0)	<0.001
Sex						0.001
Male	3,096 (57.5)	1,057 (56.6)	717 (54.3)	580 (57.7)	742 (62.2)	
Female	2,290 (42.5)	812 (43.4)	603 (45.7)	425 (42.3)	450 (37.8)	
Age						<0.001
60–69	2,698 (50.1)	1,213 (64.9)	689 (52.2)	404 (40.2)	392 (32.9)	
70+	2,688 (49.9)	656 (35.1)	631 (47.8)	601 (59.8)	800 (67.1)	
Education						0.053
Illiteracy	1,020 (18.9)	321 (17.2)	274 (20.8)	203 (20.2)	222 (18.6)	
Primary school	1,955 (36.3)	706 (37.8)	494 (37.4)	336 (33.4)	419 (35.2)	
Junior high school	1,165 (21.6)	419 (22.4)	251 (19.0)	226 (22.5)	269 (22.6)	
High school and above	1,246 (23.1)	423 (22.6)	301 (22.8)	240 (23.9)	282 (23.7)	
Insurance						<0.001
No insurance	1,049 (19.5)	419 (22.4)	256 (19.4)	185 (18.4)	189 (15.9)	
NCMS	1,603 (29.8)	537 (28.7)	427 (32.3)	292 (29.1)	347 (29.1)	
URBMI	1,168 (21.7)	364 (19.5)	272 (20.6)	217 (21.6)	315 (26.4)	
UEBMI	1,566 (29.1)	549 (29.4)	365 (27.7)	311 (30.9)	341 (28.6)	
Smoking status						<0.001
Never	3,797 (70.5)	1,296 (69.3)	965 (73.1)	728 (72.4)	808 (67.8)	
Current	755 (14.0)	308 (16.5)	170 (12.9)	118 (11.7)	159 (13.3)	
Past	834 (15.5)	265 (14.2)	185 (14.0)	159 (15.8)	225 (18.9)	
Main disease						<0.001
Circulatory system	1,697 (31.5)	553 (29.6)	497 (37.7)	331 (32.9)	316 (26.5)	
Neoplasms	1,132 (21.0)	291 (15.6)	266 (20.2)	232 (23.1)	343 (28.8)	
Injury	521 (9.7)	268 (14.3)	73 (5.5)	74 (7.4)	106 (8.9)	
Digestive system	482 (8.9)	114 (6.1)	103 (7.8)	102 (10.1)	163 (13.7)	
Respiratory system	373 (6.9)	89 (4.8)	108 (8.2)	66 (6.6)	110 (9.2)	
Other	1,181 (21.9)	554 (29.6)	273 (20.7)	200 (19.9)	154 (12.9)	
CCI score						<0.001
0	1,457 (27.1)	680 (36.4)	318 (24.1)	218 (21.7)	241 (20.2)	
1	1,450 (26.9)	482 (25.8)	418 (31.7)	276 (27.5)	274 (23.0)	
2	1,250 (23.2)	384 (20.5)	281 (21.3)	256 (25.5)	329 (27.6)	
3+	1,229 (22.8)	323 (17.3)	303 (23.0)	255 (25.4)	348 (29.2)	

*Data presented as mean ± SD or frequency (%), as appropriate. NRS 2002, The Nutritional Risk Screening 2002; BMI, body mass index; CCI, Charlson comorbidity index; no insurance (patients pay all hospital fees out of pocket); New Cooperative Medical System (NCMS; covered rural residents); Urban Resident Basic Medical Insurance (URBMI; covered urban residents without a stable job); and Urban Employee Basic Medical Insurance (UEBMI; covered employed workers). Past smokers refer to at least 6 months without smoking*.

### NRS 2002 Scores

To assess nutritional status, NRS 2002 scores were calculated (Table 1). Of the patients studied, 34.7% (1,869/5,386) showed no risk (NRS 2002 <3) after the initial screening, but 65.3% (3,517/5,386) were categorized as at risk of undernutrition. Among the patients at risk of undernutrition, Table 1 shows that 24.51% (1,320/5,386) were at moderate risk (NRS 2002 = 3); 18.66% (1,005/5,386) were at high risk (NRS 2002 = 4); and 22.13% (1,192/5,386) were at very high risk (NRS 2002 ≥ 5) and were classified as undernourished. In Table 1, the highest prevalence of undernutrition was found in patients with the disease of the circulatory system.

### Impact on Mortality, LoS, and Hospital Costs

**Figure 1:**
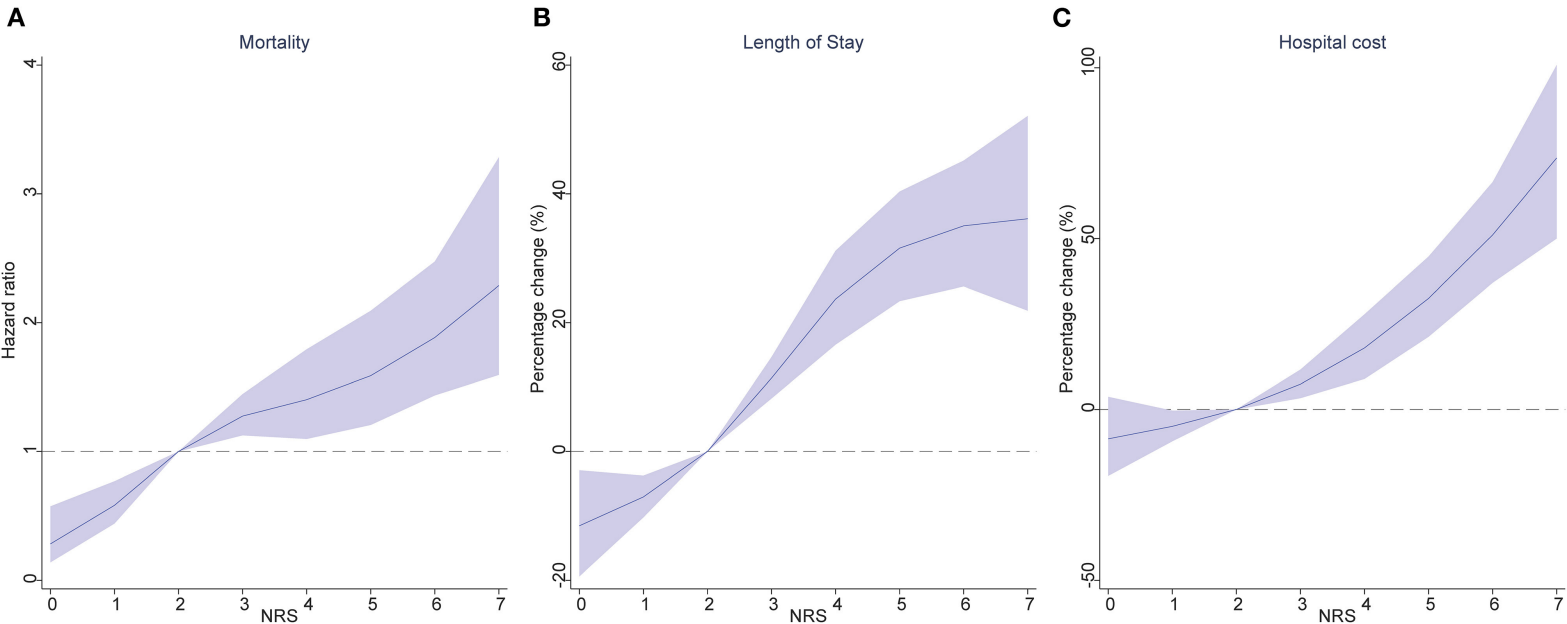
Association of the NRS 2002 score, with mortality **(A)**, length of stay **(B)**, and hospital cost **(C)**. Hazard ratios are indicated by solid lines and 95% confidence intervals by shaded areas, reference point is NRS 2002 score = 2, with knots placed at 5th, 35th, 65th and 95th percentiles), after adjusting for age, sex, education, insurance, smoking status, main disease and CCI score. NRS 2002, Nutritional Risk Screening 2002; CCI, Charlson comorbidity index.

Death occurred in 8.4% of patients (Table 1), the number of patients who died with no nutritional risk (NRS <3) was 89 (4.8%); moderate nutritional risk (NRS 2002 = 3) was 105 (8.0%); high nutritional risk (NRS 2002 = 4) was 116 (11.5%); and very high nutritional risk (NRS 2002 ≥ 5) was 143 (12.0%) (*P* < 0.001). Table 2 displays the association of nutritional risk with mortality, LoS, and hospital costs. The Cox proportional hazards model in Table 2 indicates that after adjusting for potential covariates, NRS 2002 scores = 3 (HR: 1.376, 95% CI: 1.031–1.836) were associated with a 1.5-fold higher likelihood of death; an NRS score 4 (HR: 1.982, 95% CI: 1.491–2.633) and NRS score ≥ 5 (HR: 1.982, 95% CI: 1.498–2.622) both evidenced a 2-fold higher likelihood of death compared with NRS score <3. Figure 1A displays a positive and monotonic association between the NRS 2002 score and mortality: the higher the nutritional risk, the higher the risk of death (*P* < 0.001).

**Table 2:** The association of nutritional risk with mortality, length of stay, and hospital costs.

	**Mortality, Hazard ratio (95% CI)**			**Length of stay, Percentage change (95% CI)**			**Hospital cost, Percentage change (95% CI)**		
	**Model 1^**‡**^**	**Model 2^**§**^**	**Model 3^**¶**^**	**Model 1^**‡**^**	**Model 2^**§**^**	**Model 3^**¶**^**	**Model 1^**‡**^**	**Model 2^**§**^**	**Model 3^**¶**^**
Continuous	1.226 (1.158–1.298)	1.227 (1.158–1.299)	1.215 (1.144–1.291)	8.7 (7.2–10.3)	8.5 (7–10)	8.2 (6.6–9.7)	9.4 (7.4–11.5)	9.2 (7.2–11.2)	9.2 (7.1–11.2)
**Categorical (Reference** **=** **NRS** **<** **3)**									
NRS = 3	1.566 (1.179–2.079)	1.551 (1.168–2.061)	1.376 (1.031–1.836)	16.7 (9.8–24)	16.7 (9.9–23.8)	16.4 (9.6–23.6)	19.4 (10.1–29.6)	18.7 (9.5–28.7)	17.8 (8.6–27.7)
NRS = 4	2.131 (1.611–2.820)	2.114 (1.597–2.797)	1.982 (1.491–2.633)	35.6 (26.8–45)	33.9 (25.4–43)	32.4 (24–41.4)	33.0 (21.5–45.6)	33.1 (21.7–45.5)	31.1 (19.8–43.5)
NRS ≥ 5	2.124 (1.620–2.784)	2.114 (1.611–2.774)	1.982 (1.498–2.622)	39.5 (30.8–48.8)	38.5 (30–47.5)	36.8 (28.3–45.8)	45.6 (33.6–58.7)	44.1 (32.3–56.9)	44.3 (32.3–57.4)

*NRS 2002, The Nutritional Risk Screening 2002.^**§**^Model 1: adjusted for age and sex.^**‡**^Model 2: included model 2 variables and additionally education, insurance, and smoking status. Model 3: included model 2 variables and in addition main disease, and CCI score. ^¶^Model 3: included model 2 variables and additionally main disease, and CCI score*.

The average LoS in the group with NRS scores <3 was 15.0 ± 10.7 days; NRS score 3 was 17.4 ± 15.2 days; NRS score 4 was 20.2 ± 21.6 days; and NRS scores ≥ 5 was 20.8 ± 17.5 (Table 1). Similar to the results of crude estimate analysis, after adjusting for potential covariates in the multivariable-adjusted model (Table 2), a higher NRS 2002 score 3 (percentage change: 16.4, 95% CI: 9.6–23.6), was associated with a significantly (16.4%) higher likelihood of increased LoS; NRS score 4 (32.4, 95% CI: 24–41.4) was associated with a 32.4% higher likelihood of increased LoS; and NRS score ≥ 5 (36.8, 95% CI: 28.3–45.8) was associated with a 36.8% higher likelihood of increased LoS compared with an NRS 2002 scores <3. The solid lines in Figure 1B show that the LoS increased with the NRS 2002 score.

In Table 1, the mean costs in the NRS 2002 score <3 group incurring RMB44.8 thousand (SD ± RMB45.0); NRS score 3 RMB53.5 thousand (SD ± RMB66.1); NRS score 4 RMB56.2 thousands (SD ± RMB 70.2); and NRS score ≥ 5 RMB61.9 thousand (SD ± RMB75.0). After adjusting for age, sex, education, insurance, smoking status, main disease, and CCI score covariates, Table 2 shows that the NRS score 3 group (95% CI: 8.6–27.7) was associated with a 17.8%, the NRS score 4 group (95% CI: 19.8–43.5) a 31.1%, and the NRS score ≥ 5 (95% CI: 32.3–57.4) a 44.3%, higher likelihood of increased hospital costs compared with an NRS 2002 score <3 group. Figure 1C displays the positive association between the NRS 2002 score and hospital costs, where the costs increased with nutritional risk (*P* < 0.001).

### Subgroup Covariate Analysis

#### Mortality

**Figure 2:**
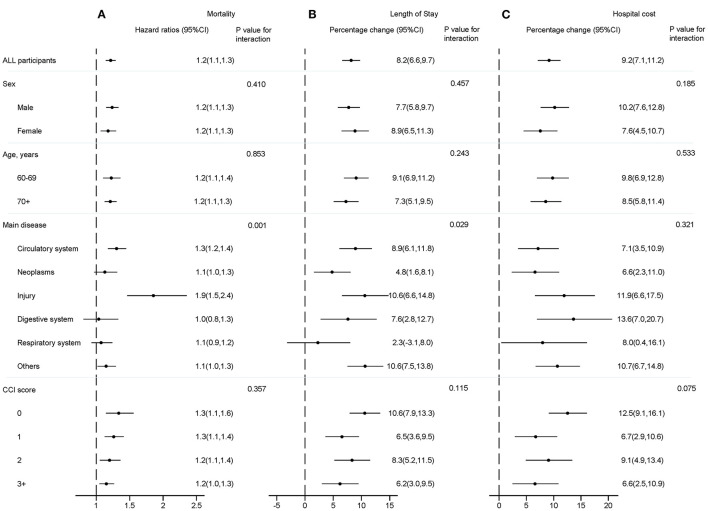
Hazard ratios, percentage change and 95% CI for the association between the NRS 2002 score and mortality **(A)**, length of stay **(B)**, and hospital cost **(C)** adjusting for age, sex, education, insurance, smoking status, main disease and CCI score. CCI, Charlson comorbidity index; CI, confidence interval.

**Figure 3:**
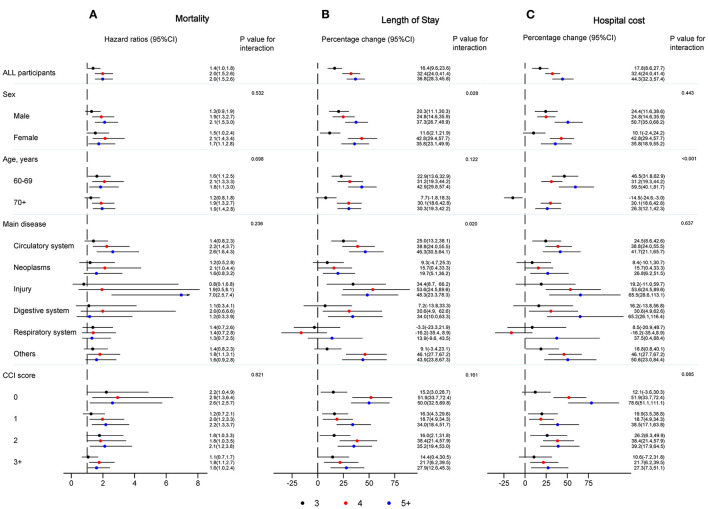
Hazard ratios, percentage change and 95% CI for the association between NRS 2002 categories and mortality **(A)**, length of stay **(B)**, and hospital cost **(C)**, with NRS 2002 <3 as a reference group, adjusting for age, sex, education, insurance, smoking status, main disease, and CCI score. CCI, Charlson comorbidity index; CI, confidence interval, NRS 2002, Nutritional Risk Screening 2002.

For the analysis of overall mortality, Figure 2A shows the association between continuous NRS 2002 score and mortality among those with injury and cardiovascular system diseases was stronger than that among those with neoplasms, and diseases of the digestive system, respiratory system, and “other” diseases (*P* = 0.001), but did not differ by sex (*P* = 0.410), age (*P* = 0.853), and CCI score (*P* = 0.357). In addition, the relative risk of death from all causes was most notable among immobile older patients with injury (HR: 1.9, 95% CI: 1.5–2.4; *P* < 0.001). Also, in Figure 3A, the association of categorical NRS 2002 scores with mortality did not differ by sex, age, disease, and CCI score, although it appeared stronger in the diagnosis of cardiovascular system diseases, injury, and among CCI scores of 0 and 1.

#### Length of Stay

Concerning LoS (Figure 2B), the association between continuous NRS 2002 scores and LoS among those with diseases of the cardiovascular and digestive system, neoplasms, injury, and “other” diseases was stronger than that among those with diseases of the respiratory system, but did not differ by sex, age, and CCI score. The percentage change was most notable among immobile older inpatients with injury (10.6, 95% CI: 6.6–14.8; *P* < 0.05). In Figure 3B, the association of categorical NRS 2002 scores with LoS additional differ by sex, but not by age and CCI score, although it appeared stronger among patients aged 60–69 years old, and among CCI scores of 0 and 2.

#### Hospital Costs

As can be seen in Figure 2C, there was no significant association between continuous NRS 2002 scores and hospital costs, although it appeared stronger among men, among younger older patients, among those with diseases of the digestive system, injury, and “other” diseases, and among the CCI score of 0. The percentage change was most notable among immobile older inpatients with digestive diseases (13.6, 95% CI: 7.0–20.7; *P* > 0.05). In Figure 3C, the association of categorical NRS 2002 scores differ by age, while the association appears stronger among men, among those with injury, diseases of the digestive system, and “other” diseases, and among CCI scores of 0.

## Discussion

This national study is among the first to examine the burden of undernutrition in Chinese older immobile inpatients with diseases. After adjustment for covariates, nutritional risk, measured by NRS 2002, negatively impacted mortality and hospital LoS and increased the cost of hospitalization.

The prevalence of undernutrition risk, based on a positive NRS 2002 result (score ≥ 3), was 65.3% in this study. Our NRS result was the same as in Switzerland (64.6 and 62.7%) (28), but significantly higher than estimates for Denmark (23%) (35) and Brazil (48.1%) (36). The difference between the prevalence of nutritional risk reflects different nutritional risk assessment tools, inclusion and exclusion criteria used, and the effect of local factors, including the health system characteristics in different countries and the standards of medical treatment received (3, 4, 28, 37–40).

The association between nutritional risk and mortality has been known for some time (4, 7, 8), and our mortality rate of 8.4% saw marked differences between NRS 2002 groups (*P* < 0.001). A Swiss study of 2028 patients hospitalized in medical wards reported that nutritional risk assessed by NRS 2002 at the time of hospital admission was a good predictor of short-term (30 and 180 days) mortality, with an increased risk in mortality comparing patients scoring NRS 2002 scores of 3 with those with ≥ 5 points (8). These findings are in line with our results, which also show an increased risk of mortality between the NRS 2002 scores of 3 and 5+ (HR: 1.376 vs. HR: 1.982). Our results suggest that the increase in nutritional risk may be a sign of the approach of life's end among older immobile inpatients. Considering the effect of nutritional support interventions on clinical outcomes (11, 18, 41), maintaining nutritional status would be beneficial for survival among older immobile inpatients, even for those with low nutritional risk.

We also examined the association of nutritional risk and mortality, LoS, and hospital costs between different demographic characteristics, diseases, and comorbidity subgroups. Overall, we found little variation within these groups. The association between nutritional risk and mortality was not substantially different in men compared to women, 60- to 69-year-olds compared to 70+-year-olds, smokers, education level, type of insurance, and different CCI scores, suggesting that undernutrition is a risk factor across the entire immobile older inpatients population. In line with previous studies, cardiovascular system disease and injury influenced the association of nutritional risk with mortality (3, 42, 43). In the subgroup analysis, we also found that those with baseline CCI scores 0 and 1 had higher mortality related to NRS 2002 scores of 4+ compared to those with CCI scores ≥ 2 points. Those with baseline CCI scores of 0, 1, and 2 had higher mortality related to NRS 2002 scores of 5+ compared to those with CCI scores ≥ 3 points. However, the associations of nutritional risk with mortality in our study did not substantially change after adding these comorbid diseases to the models. Therefore, screening and treatment of undernutrition should not be limited to patient populations with specific illnesses, but should include all hospitalized older patients. Importantly, we observed that sex influenced the association of NRS 2002 scores of 5+ with mortality, with men experiencing a higher risk of death than women (44, 45).

Specifically, subgroup analysis indicated that the most notable mortality-specific comorbidities and LoS-specific comorbidities were injuries, while the most notable cost-specific comorbidities were the diseases of the digestive system. One explanation is that approximately half of the participants were aged 70 years and older, and these patients may be unable to withstand surgical stresses, therefore patients undergoing surgical treatment due to injuries (such as fractures and peripheral nerve injuries) might increase the risk of medical complications, longer LoS, and death (46, 47). Furthermore, gastric cancer and inflammatory bowel disease are the main causes of digestive diseases among the elderly in this study, which place a significant financial burden on families of patients and the healthcare systems because of its chronicity and need for expensive therapies and surgery (48, 49).

The average LoS in patients with NRS 2002 scores <3 was shorter than the other groups, and a statistically significant association between LoS and a positive NRS 2002 result was demonstrated in our multivariate analysis. Similar LoS findings have been reported in the United States (50), Switzerland (8), and Singapore (7), and an observational cohort study conducted in Colombia (16) reported that undernutrition at admission was independently associated with a further 1.43 LoS days after controlling for socioeconomic characteristics, disease-related factors, and medical or nursing interventions. However, the average LoS in the current study was longer than previous reports (7, 8, 16, 50), which reflects that our participants were immobile.

The association between nutritional risk and LoS was significantly different for patients with cardiovascular and digestive system diseases, neoplasms, and injury, a finding also in accordance with previous studies (3, 8, 28). Therefore, our study strengthens the recommendation of the European Society of Parenteral and Enteral Nutrition (ESPEN) that nutritional risk screening should be performed for all hospitalized patients. In the absence of adequate screening capacities, we recommend hospitals focus on patients with cardiovascular and digestive system diseases, cancer, and injury to promote medical decision-making, save medical and nursing resources, and shorten the LoS. We also found evidence for a differing NRS 2002 scores of ≥ 5-LoS association between men and women, perhaps due to the sex differences in metabolic regulation among the older population, and biological sex impact on the pathogenesis of numerous diseases, such as the metabolic disorders, which is a nutritional challenge and affects clinical outcomes (51, 52).

Besides the clinical outcomes, international studies have revealed the increased hospital costs and overall economic burden associated with undernutrition in hospitals. In China, there is limited up-to-date information regarding the hospitalization costs associated with hospital undernutrition. After covariate adjustment, “at-risk” (NRS 2002 scores of 3+) older patients had higher hospital costs compared with “not-at-risk” patients (NRS 2002 scores <3). This is consistent with previous studies that reported that undernutrition could raise by 30.13% the average cost associated with hospitalization (16). Similar hospital costs findings have also been reported in Brazil (53), where the mean daily cost of care was 61% higher for the undernourished compared to well-nourished patients among 25 Brazilian hospitals.

Several studies found that early nutrition intervention for “at-risk” patients is highly cost-effective compared to delayed nutrition therapy (54–56). We recommend improved nutritional management of nutritionally “at-risk” older inpatients, for example, by issuing institutional guidelines and implementing more thorough training and enhanced collaboration between physicians, nurses, and dieticians. Developing a nutritional risk information reporting system in the HIS, which automatically notifies the clinical nutrition department to the presence of “at-risk” patients, would improve the quality of hospital care, optimize medical and nursing resources, and economize on hospital costs.

One limitation of our study was a follow-up for 90 days, with future investigations recommended undertaking observations over a longer duration to better clarify the present findings. In addition, our use of a limited number of nutritional assessment tools restricted the comparison of our results with other studies. Since our study was the first of its kind focusing on immobile older inpatients in China, few comparisons could be made to other Chinese studies. As discussed above, the participants were immobile, but the mandatory NRS 2002 tool does not include this component. We also do not have data on whether the nutritional status might worsen during the hospital stay, which might impact the evaluated health outcomes. Future Chinese studies should employ a wider range of evaluations and further assess the clinical and economic impact of nutritional interventions (such as nutritional screening and treatment) in preventing undernutrition across the different Chinese health settings. Also, future studies should develop a more detailed classification of hospital costs specifically associated with being nutritionally “at-risk.” It will be of interest to further assess the different types of health expenditures, namely, the parenteral nutrition, enteral nutrition, medical treatments, nursing care, and X-rays, among nutritionally “at-risk” patients in China.

## Conclusions

Early assessment, identification, and adequate management of “at-risk” undernutrition patients are warranted. Considering nutritional support can improve health outcomes and reduce healthcare costs. Greater attention to nutrition during the hospital stay and post-discharge among the older population is necessary to provide enhanced quality interventions and care for this vulnerable subpopulation.

## Data Availability Statement

The raw data supporting the conclusions of this article will be made available by the authors, without undue reservation.

## Ethics Statement

The studies involving human participants were reviewed and approved by Peking Union Medical College Hospital (S-700). The patients/participants provided their written informed consent to participate in this study.

## Author Contributions

Study concept and design by XWu. Analysis, interpretation of data, editing of the manuscript, and drafting of tables by HL and DZ. A critical review of the manuscript for important intellectual content by XWu, HL, DZ, SN, and EM. Patient recruitment, data collection, and manuscript editing by BS, JJ, YL, XWe, SC, WC, SN, and EM. All authors critically reviewed and approved the manuscript before it was submitted.

## Funding

This work was supported by the National Health and Family Planning Commission (Beijing, China) (Grant Number: 201502017). The funding bodies had no specific role in study design or data collection, analysis, and interpretation, or manuscript conception and writing.

## Conflict of Interest

The authors declare that the research was conducted in the absence of any commercial or financial relationships that could be construed as a potential conflict of interest.

## Publisher's Note

All claims expressed in this article are solely those of the authors and do not necessarily represent those of their affiliated organizations, or those of the publisher, the editors and the reviewers. Any product that may be evaluated in this article, or claim that may be made by its manufacturer, is not guaranteed or endorsed by the publisher.

## Abbreviations

NRS 2002: The Nutritional Risk Screening 2002
BMI: body mass index
CCI: Charlson comorbidity index.
